# Genetic Testing Unveils a Novel Thrombospondin‐1 Domain Containing Protein 1 Gene Variant as the Cause of Chronic Edema in a 79‐Year‐Old Woman

**DOI:** 10.1111/cge.70247

**Published:** 2026-09-09

**Authors:** Eiko Amo, Takafumi Toyohara, Naoya Saijo, Satoko Sato, Jun Takayama, Yoshiko Suto, Yuji Noguchi, Sosuke Kinomura, Yusuke Ishizuka, Shun Watanabe, Koichi Kikuchi, Rui Makino, Mai Yoshida, Yuji Oe, Tasuku Nagasawa, Koji Okamoto, Takehiro Suzuki, Mariko Miyazaki, Shinya Kawaguchi, Takashi Suzuki, Takaaki Abe, Shigeo Kure, Atsuo Kikuchi, Tetsuhiro Tanaka

**Affiliations:** ^1^ Department of Nephrology and Hypertension Tohoku University Hospital Sendai Japan; ^2^ Department of Pediatrics Tohoku University Graduate School of Medicine Sendai Japan; ^3^ Department of Pathology Tohoku University Hospital Sendai Japan; ^4^ Department of Rare Disease Genomics Tohoku University Graduate School of Medicine Sendai Japan; ^5^ Department of AI and Innovative Medicine Tohoku University Graduate School of Medicine Sendai Japan; ^6^ Tohoku Medical Megabank Organization Tohoku University Sendai Japan; ^7^ Department of Medical Science Tohoku University Graduate School of Biomedical Engineering Sendai Japan; ^8^ Iwaki Yumoto Hospital Iwaki Japan; ^9^ Miyagi Children's Hospital Sendai Japan

**Keywords:** chronic edema, elderly, genetic testing, thrombospondin‐1 domain containing protein 1 gene

## Abstract

Edema requires management tailored to its underlying etiology; however, in some cases the cause remains elusive. We describe a 79‐year‐old woman with lifelong unexplained peripheral edema. Comprehensive evaluation excluded common etiologies such as heart failure, renal dysfunction, and venous thrombosis. Whole‐genome sequencing identified a novel homozygous splice‐site variant (NM_018676.4:c.58+2T>G) in the *thrombospondin‐1 domain–containing protein 1* (*THSD1*) gene, which has previously been associated with non‐immune hydrops fetalis (NIHF). This report describes, to our knowledge, the first elderly patient with chronic peripheral edema harboring a likely pathogenic THSD1 variant, suggesting that THSD1‐related disease may, in rare instances, persist beyond the perinatal period and present into late adulthood. Although causality cannot be established definitively from a single case, the findings highlight the potential utility of genetic testing in adults with chronic unexplained edema.

## Graphical abstract

A 79‐year‐old woman with lifelong peripheral edema and an affected sister was found to harbor a novel homozygous THSD1 splice‐site variant. Reduced THSD1 expression in dermal endothelial cells supported the possibility that this variant contributes to chronic hereditary edema.

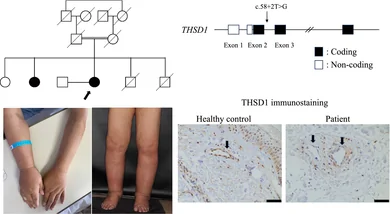

## 1 Introduction

Lower limb edema is a common and clinically important symptom, as it may signal underlying systemic diseases. Common causes include heart failure, renal dysfunction, liver disease, thyroid disorders, hypoalbuminemia, and medication use [1]. In patients over 50 years of age, leg edema is most frequently attributed to chronic venous insufficiency [2], whereas in fetuses with hydrops, congenital cardiovascular anomalies and chromosomal abnormalities are often implicated [3]. Since the management of peripheral edema depends on its underlying cause, accurate diagnosis is essential. A range of diagnostic tests—including urinalysis, blood tests, electrocardiography, chest radiography, echocardiography, abdominal ultrasonography, lower limb Doppler ultrasonography, and computed tomography–are routinely employed to identify the etiology.

Some forms of edema are congenital. Hydrops fetalis typically presents in the fetal period and is diagnosed by antenatal ultrasonography and genetic testing [4]. When hydrops is secondary to cardiovascular or hematologic disorders, management targets the underlying disease; however, therapeutic intervention is challenging when no causative condition is identified. Prognosis is variable and depends on the specific genetic variant [5]; some cases result in fetal demise, whereas others resolve spontaneously.

A representative genetic cause involves the *thrombospondin‐1 domain–containing protein 1* (*THSD1*) gene. *THSD1* encodes a transmembrane protein with a thrombospondin Type 1 domain and is predominantly expressed in hematopoietic and endothelial cells [6]. Homozygous *THSD1* variants have been associated with non‐immune hydrops fetalis (NIHF) in infants, as well as congenital heart disease, hemangiomas, prematurity, and embryonic lethality [7]. Hydrops fetalis due to *THSD1* mutations is typically identified in the perinatal period.

Here, we report a case of an elderly female who had experienced recurrent limb edema of unknown origin for over 60 years. She was admitted to our hospital for further evaluation. Notably, her sister had a similar clinical history, prompting suspicion of a hereditary condition. Whole‐genome sequencing revealed a novel variant in the *THSD1* gene. To our knowledge, this is the first report of a *THSD1* mutation presenting as peripheral edema in adulthood. This case highlights the potential utility of genetic testing in uncovering the etiology of unexplained edema even in elderly patients, thereby avoiding unnecessary diagnostic procedures and enabling symptom‐focused, precise clinical management.

## 2 Case Report

A 79‐year‐old woman was referred to our hospital for evaluation of chronic upper and lower extremity edema present since early childhood. An initial workup at our institution during adolescence failed to identify a definitive etiology. Over subsequent decades, the edema persisted and was complicated by recurrent episodes of cellulitis, each requiring antibiotic therapy. Her medical history included essential hypertension managed with a calcium channel blocker, Ménière's disease, and prior surgical treatment for bowel obstruction. She had no history of aneurysmal disease or hemorrhagic/bleeding disorders. She was born to consanguineous parents (first cousins), and one sister also exhibited chronic peripheral edema (Figure 1A), raising suspicion for a hereditary condition. Prior to referral, she was admitted to a local hospital with cellulitis and severe lower limb edema, which resulted in impaired mobility, profound fatigue, and dysphagia. Laboratory evaluation revealed marked hypoalbuminemia (serum albumin 1.7 g/dL), while cardiac dysfunction, hepatic impairment, venous thrombosis, and proteinuria were excluded. Following antibiotic therapy and diuretic treatment with furosemide, her edema improved and she was discharged. However, because the underlying cause remained unclear, she was subsequently referred to our hospital for further investigation.

**FIGURE 1:**
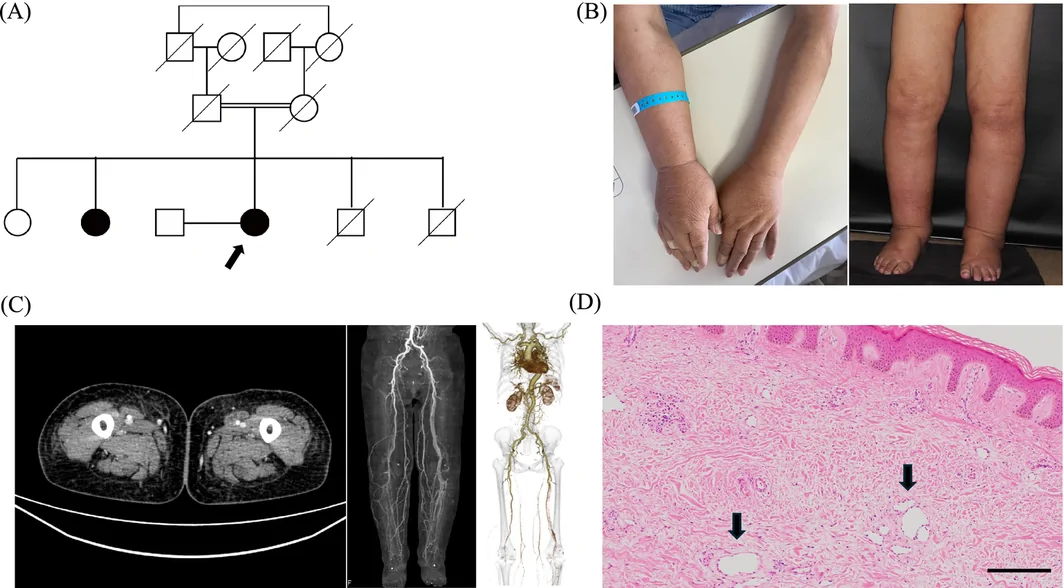
(A) Pedigree chart spanning three generations, demonstrating consanguineous marriage. (B) Clinical appearance of peripheral edema involving both the upper and lower extremities. (C) Contrast‐enhanced computed tomography images of the lower extremities. (D) Histopathological findings of a skin biopsy. Arrows indicate markedly dilated lymphatic vessels (scale bar: 200 μm). [Colour figure can be viewed at wileyonlinelibrary.com]

On admission to our hospital, her vital signs were stable: body temperature 36.0°C, blood pressure 139/75 mmHg, pulse 74 beats per minute, body weight 61.4 kg, and height 153.8 cm. Physical examination revealed severe pitting edema involving both the upper and lower extremities (Figure 1B). Laboratory tests demonstrated hypoalbuminemia (serum albumin 3.0 g/dL), partially improved compared with the prior measurement, likely reflecting recovery from cellulitis. C‐reactive protein and urinary protein levels were within normal limits, and additional hematological and biochemical findings are summarized in Table 1. Chest radiography showed no cardiomegaly or pleural effusion. Electrocardiography was unremarkable. Transthoracic echocardiography demonstrated preserved left ventricular systolic function (ejection fraction, 63%) with mild left atrial enlargement and mild mitral and pulmonary regurgitation; these findings were hemodynamically insignificant and unlikely to account for the patient's edema. Lower extremity venous ultrasonography and contrast‐enhanced computed tomography excluded deep venous thrombosis (Figure 1C). Small‐bowel endoscopy was performed to evaluate for protein‐losing gastroenteropathy and revealed no evidence of enteric protein loss. Skin biopsy from the edematous region demonstrated dilated lymphatic vessels, consistent with lymphatic edema (Figure 1D).

**TABLE 1:** Hematological and biochemical findings.

Variable	Reference range, adults	On presentation, this hospital
*Blood*		
Hemoglobin (g/dL)	11.6–14.8	11.1
Hematocrit (%)	35.1–44.4	35.0
Red‐cell count (per mm^3^)	3 860 000‐4 920 000	3 750 000
White‐cell count (per μL)	3300‐8600	3700
*Differential count* (%)		
Neutrophils	38.5–80.5	42.1
Lymphocytes	16.5–49.5	46.9
Monocytes	2.0–10.0	7.5
Eosinophils	0.0–8.5	2.7
Basophils	0.0–2.5	0.8
Platelet count (per μL)	158–348	234 000
Sodium (mmol/l)	138–145	141
Potassium (mmol/l)	3.6–4.8	3.9
Chloride (mmol/l)	101–108	108
Calcium (mg/dL)	8.8–10.1	8.6
Magnesium (mg/dL)	1.8–2.4	2.0
Urea nitrogen (mg/dL)	8–20	22
Creatinine (mg/dL)	0.46–0.79	0.84
eGFR (mL/min/1.73m^2^)	60 ≦	50
Glucose (mg/dL)	73–109	85
Total protein (g/dL)	6.6–8.1	5.9
Albumin (g/dL)	4.1–5.1	3.0
Alanine aminotransferase (U/l)	7–23	14
Aspartate aminotransferase (U/l)	13–30	25
N‐terminal pro‐B‐type natriuretic peptide (pg/mL)	≦ 18.4	16.7
Erythrocyte sedimentation rate (mm/h)	3–15	24
C‐reactive protein (mg/l)	≦ 0.14	0.04
*Urine*		
Bilirubin	Negative	Negative
Blood	Negative	+−
Clarity	Negative	Negative
Color	Yellow	Yellow
Glucose	Negative	Negative
Ketones	Negative	Negative
pH	5.0–7.5	5.5
Protein		Negative
Erythrocytes (per high‐power field)		1–4
Specific gravity	1.005–1.030	1.014
Urobilinogen		+−
Leukocytes (per high‐power field)		< 1

Given the family history of edema, the persistence of symptoms since childhood, and parental consanguinity, a hereditary disorder was suspected. With the patient's informed consent, whole‐genome sequencing was performed, which identified a novel homozygous splice‐site variant in *THSD1* (NM_018676.4:c.58+2T>G) (Figure 2A). Whole‐genome sequencing data were also interrogated for established genes associated with primary lymphedema and lymphatic anomalies, including *FLT4* (*VEGFR3*), *FOXC2*, *GATA2*, *CCBE1*, and *PIEZO1*. No pathogenic or likely pathogenic variants that could explain the patient's phenotype were identified in these genes. The *THSD1* variant was absent from ToMMo 61KJPN and gnomAD v4.1.0. According to the 2015 ACMG/AMP framework, with refinement of the PVS1 strength based on ClinGen SVI guidance, the variant was classified as “likely pathogenic” (PVS1_Strong, PM2). PVS1 was applied at the Strong rather than Very Strong level because the variant is located near the extreme 5′ end of the transcript, where downstream translational re‐initiation or residual protein function cannot be completely excluded. SpliceAI strongly predicted loss of the canonical donor splice site (donor loss Δ score = 0.97). Notably, a previous report described non‐immune hydrops fetalis in a consanguineous family caused by a pathogenic *THSD1* variant [7]. Although the present variant has not been previously documented, its nature as a splice‐site variant strongly suggests pathogenicity.

**FIGURE 2:**
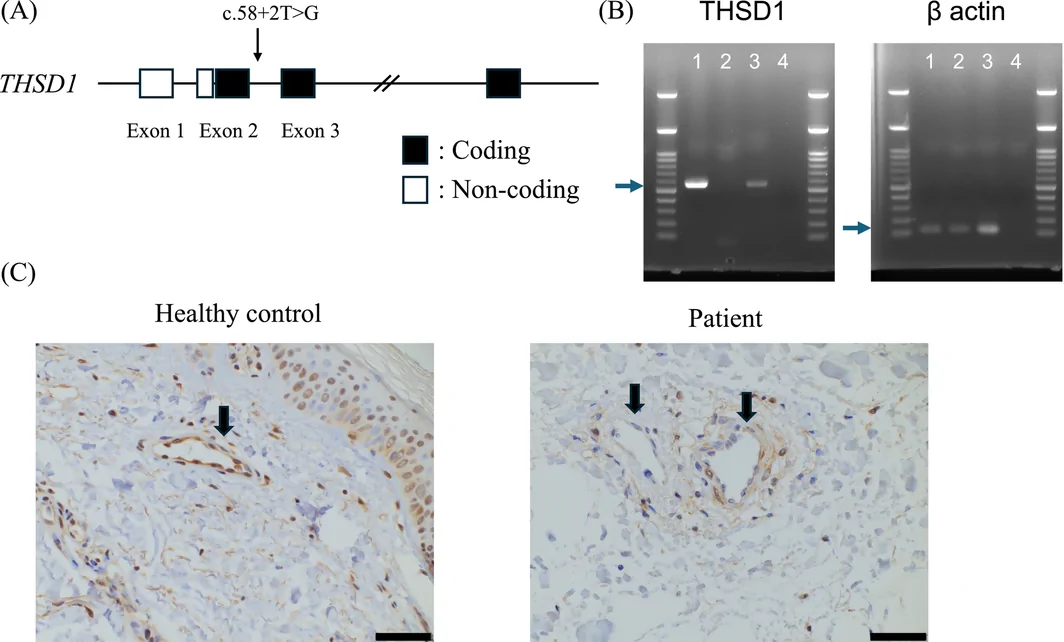
(A) Schematic representation of the THSD1 gene mutation identified in this patient. (B) THSD1 expression in urinary sediment from a healthy control and the patient. RT‐PCR was performed using RNA extracted from urinary sediment obtained from a healthy control (lane 1) and the patient (lane 2). Human kidney cDNA (Human Kidney Marathon‐Ready cDNA) was included as a positive control (lane 3), and H_2_O was included as a negative control (lane 4). RNA samples from the healthy control and the patient were processed under identical conditions to permit comparison of THSD1 transcript detection. The following primers were used: THSD1 Forward, 5′‐GATGCTTGGCGCTCTACCTC‐3′; THSD1 reverse, 5′‐GACAGGCCATTCCACCTTCA‐3′ (expected product size, 564 bp); β‐Actin forward, 5′‐CAATGTGGCCGAGGACTTTG‐3′; and β‐Actin reverse, 5′‐CATTCTCCTTAGAGAGAAGTGG‐3′ (expected product size, 126 bp). The THSD1 forward primer was designed within the distal region of exon 1, and the reverse primer was designed within exon 3. PCR amplification was performed using KOD FX Neo DNA polymerase (TOYOBO, Osaka, Japan). (C) Immunohistochemical staining of skin biopsy tissue using an anti‐THSD1 antibody. Arrows indicate dermal capillary endothelial cells with reduced THSD1 expression in the patient compared to a healthy control (scale bar: 50 μm). [Colour figure can be viewed at wileyonlinelibrary.com]

To further investigate, we performed RT‐PCR using RNA extracted from the patient's urinary sediment. The expected THSD1 transcript spanning the variant site was not detected, providing supportive molecular evidence consistent with disrupted mRNA processing or markedly reduced transcript abundance in the region surrounding the variant (Figure 2B). Furthermore, immunohistochemical staining of skin biopsy tissue was performed using an anti‐THSD1 antibody (Atlas Antibodies). In dermal capillary endothelial cells, THSD1 protein expression appeared reduced compared with control tissue (Figure 2C). Taken together, the clinical, genetic, and pathological findings suggest that the THSD1 variant was likely associated with the patient's lifelong edema.

## 3 Discussion

To our knowledge, this is the first reported case of primary lymphedema in an adult associated with a *THSD1* mutation. Lymphedema results from the accumulation of protein‐rich fluid in the interstitial space due to impaired lymphatic drainage. Primary lymphedema is idiopathic or congenital, whereas secondary is associated with malignancy, trauma, filariasis, or iatrogenic causes. In adults, primary lymphedema is usually idiopathic and rare (< 1% of all lymphedema cases) [8]. This patient manifested a phenotype distinct from previously described THSD1‐variant cases. Pathogenic variants in THSD1 have been associated with NIHF, typically identified in the fetal period, in which the predominant manifestation is pleural effusion rather than peripheral edema [9]. However, in this case, hydrops fetalis was not observed prenatally. In addition, the patient experienced persistent limb edema of unknown mechanism for more than 60 years.

*THSD1* encodes thrombospondin Type 1 domain–containing protein 1, which is expressed in vascular endothelial cells and contributes to endothelial adhesion, extracellular matrix interactions, and vascular stability [10]. Previously reported pathogenic *THSD1* variants include c.617G>A (p.Cys206Tyr), c.670G>A (p.Arg224Ter), and c.892G>T (p.Glu298Ter) [5, 11]. In the present case, we identified a novel splice‐site variant, c.58+2T>G, which is suggestive of pathogenicity according to ACMG/AMP criteria. Although the precise aberrant splice product could not be identified, the c.58+2T>G variant affects the canonical +2 donor splice position, which is critical for normal pre‐mRNA splicing. SpliceAI strongly predicted loss of the canonical donor splice site (donor loss Δ score = 0.97). Disruption of this site would be expected to cause aberrant splicing, potentially resulting in exon skipping, intron retention, or activation of a cryptic splice site. Such abnormalities could lead to a frameshift, premature termination, and nonsense‐mediated decay, thereby reducing THSD1 expression. However, because the variant is located near the extreme 5′ end of the transcript, downstream translational re‐initiation or residual protein function cannot be completely excluded. Accordingly, PVS1 was conservatively applied at the Strong rather than Very Strong level. Consistent with a splicing defect, our RT‐PCR findings suggested disrupted mRNA processing or markedly reduced transcript abundance in the region surrounding the variant. Nevertheless, the relatively mild, non‐lethal phenotype in this patient raises the possibility that some residual transcript or protein function may be preserved. This interpretation remains speculative and warrants further transcript‐level investigation.

Deficiency or dysfunction of *THSD1* is thought to impair endothelial integrity, resulting in increased vascular permeability and subsequent interstitial fluid leakage. In addition, reports of *THSD1*‐related NIHF suggest that abnormal lymphatic development or function may also play a role, leading to impaired lymphatic drainage and chronic lymphedema [9]. In this patient, histological examination revealed dilated lymphatic vessels, and immunohistochemistry demonstrated reduced THSD1 expression in dermal capillary endothelial cells, supporting both increased vascular permeability and lymphatic dysfunction as potential contributors to the phenotype. Thus, *THSD1* variants may cause edema through a dual mechanism involving vascular barrier dysfunction and lymphatic insufficiency.

This report has several limitations. First, it describes a single patient and therefore cannot establish definitive causality, prevalence, or the full phenotypic spectrum of THSD1‐related disease. Second, although the family history and parental consanguinity support a recessive hereditary disorder, segregation analysis could not be performed because the affected sister was no longer available for evaluation. Third, although RT‐PCR using RNA extracted from the patient's urinary sediment failed to detect the expected THSD1 transcript spanning the variant site, the precise aberrant transcript could not be identified or sequenced, and no minigene assay was performed. Therefore, the specific molecular consequence of the c.58+2T>G variant remains incompletely defined. Finally, detailed longitudinal and quantitative assessments of edema severity and lymphatic function were not available. Nevertheless, the lifelong phenotype, rarity and predicted splice‐disrupting nature of the variant, recessive inheritance pattern, histological evidence of lymphatic edema, absence of the expected THSD1 transcript spanning the variant site, and reduced THSD1 immunostaining collectively support the interpretation that this variant is the most plausible explanation for the patient's clinical presentation.

This case highlights the diagnostic value of genetic testing in adults with unexplained edema. A previous study reported a *fibronectin 1* variant in an elderly patient with edema due to fibronectin glomerulopathy [12]. Our findings similarly indicate that long‐standing edema in adults may have a genetic basis. As genomic testing becomes more accessible, we propose incorporating genetic analysis into the diagnostic work‐up of adults with persistent or atypical edema.

## Funding

This work was supported by Astellas Pharma and AMED Moonshot Research and Development Program grants.

## Consent

This study was conducted according to the principles of the Declaration of Helsinki. The patient was given oral explanations and gave written consent in advance.

## Conflicts of Interest

The authors declare no conflicts of interest.

## Data Availability

Raw data were generated at Tohoku University and derived data supporting the findings of this study are available from the corresponding author on request. The *THSD1* variant NM_018676.4.58+2T>G has been submitted to ClinVar (accession number: SCV007666058).
